# Cost-Effectiveness and the Distinction Between Quantitative and Qualitative Disability Discrimination

**DOI:** 10.1007/s11673-025-10431-w

**Published:** 2025-07-22

**Authors:** Lasse Nielsen

**Affiliations:** 1 Philosophy, University of Southern Denmark, Campusvej 55, 5230 Odense M, Denmark; 2 Centre for the Experimental-Philosophical Study of Discrimination, Political Science, Aarhus University, Bartholins Allé 7, 8000 Aarhus C, Denmark

**Keywords:** Cost-effectiveness, Disability, Discrimination, Frances Kamm, Samuel Kerstein, Impartial interest

## Abstract

Since standard measures of health effect ascribe negative value to disabilities, it is commonly believed that a cost-effective scheme for allocation of healthcare resources discriminates against people with disabilities. It is still a question for discussion, however, when and why such discrimination is justified. In this paper I account for the central normative substance of this disability discrimination problem, and I defend the claim that it is more justifiable to discriminate against disabled people based on lifespan considerations than on assessments of their reduced quality of life. I term this *the asymmetry intuition*. Based on some prior attempts to explain the asymmetry intuition, I offer *the Reasonable Impartial Interest Argument* as the best possible way to defend it. If my argument is sound, this moves us a step further towards a cost-effective priority setting that does not unjustly discriminate against people with disabilities.

## Introduction

There is an increasing demand for healthcare priority setting and rationing between competing healthcare needs (Bognar and Hirose 2014; Ubel et al. 1996). Since rationing decisions affect important interests, these decisions should be morally justified. It is generally accepted that morally justified healthcare rationing must be non-discriminatory and cost-effective (Baron 1995; Rawlins and Dillon 2005; Ubel 2001, 182; Bognar 2010; Lippert-Rasmussen 2023). However, given conventional concepts of cost-effectiveness, resources spent on treatment to people with disabilities (for conditions not related to their disability) are often spent less cost-effectively, ceteris paribus, than if spent on similar treatment to non-disabled people (Bognar 2020; Lippert-Rasmussen 2023). Thus, it is reasonable to conclude that a cost-effective allocation of healthcare resources discriminates against people with disabilities. Call this *the disability discrimination problem*.

To make progress in solving the disability discrimination problem we need a better understanding of the ethics involved. We need careful elaboration of the ethical values of cost-effectiveness and non-discrimination to theoretically strengthen our judgements on when and why discrimination against disabled people is justified. This is the aim of this paper. In the following section, I account for the disability discrimination problem in some detail. The next section introduces a distinction between quantitative (in reference to lifespan) and qualitative (in reference to life quality) disability discrimination, and I argue that the former is more morally justified than the latter. This, I conclude, sheds new light on the justifiability of disability discrimination in healthcare priority setting.

## The Disability Discrimination Problem

To understand the disability discrimination problem in more detail, we need to account for its central normative parts. At its most foundational level, the problem displays a conflict between the abstract values of fairness, understood as treating people equally, and beneficence, understood as doing good for others (Beauchamp and Childress 2001). Applied to basic healthcare entitlements of people with disabilities, this conflict is reiterated as a conflict between cost-effectiveness and disability discrimination at the level of priority setting.

By *cost-effectiveness*, I understand the ideal of spending resources most effectively for the purpose of maximizing health benefit (Hadorn 1992; Menzel 1992; Singer et al. 1995; Stein 2004; Bognar 2020). A cost-effective healthcare prioritization seeks to produce as much health benefit as possible with a given bundle of resources or, alternatively, to produce a given health benefit at the lowest possible cost. Cost-effectiveness is thus a rationing-sensitive and maximizing extension of the value of beneficence, where doing good for others is expanded into maximizing possible benefits for others, taking cost into consideration. By this definition, I am treating cost-effectiveness as a fundamental and abstract normative principle, and I am deliberately setting aside how cost-effectiveness analysis is used as a tool in practice.

There are some relevant differences here between theory and practice. First, cost-effectiveness analysis rarely (if ever) uses absolute QALY (quality-adjusted life year) estimates. Instead, it usually compares the additional value (per cost) of one type of treatment over another for the same condition for the same group of patients. Second, in practice, cost-effectiveness ratios are determined based on generic measures and large-scale data, using averages to account for health-related quality of life (Bognar 2020). Third, cost-effectiveness is typically framed as a threshold-concept (although the threshold is often more like a range) and the calculations are intended to test if treatments meet the healthcare system’s standards, in which case they will be provided equally for all types of patients. In effect, the measures do not directly track differences between patients with and without disabilities.

Given this, the disability discrimination problem seems to target cost-effectiveness at the abstract level, not how it’s actually used. Does this imply that the discussion of the disability-discrimination problem is a non-starter? That the philosophical concern with cost-effectiveness being discriminatory stem from a misunderstanding of health economics? Some ethicists seem to imply something like this. Bognar, for example, claims that the objections to cost-effectiveness analysis based on disability discrimination make the fallacy of merely showing that cost-effectiveness could *possibly* be used to discriminate, from which they mistakenly draw the conclusion that it is *actually* used to discriminate (Bognar 2020).

But this dismissal is too quick. There are good reasons to be concerned. First, although cost-effectiveness analysis is rarely intended to discriminate, it can be used in ways that indirectly discriminate against people with disabilities. This is the case, for example, if medicines for conditions that differentially affect disabled people are deprioritized (Watermeier and Swarts 2023). Second, even when cost-effectiveness analysis is used in ways that do not involve discrimination against people with disabilities, we still need to investigate the problem theoretically. This is because cost-effectiveness *as a principle* is discriminatory, even when cost-effectiveness analysis *as it is used in practice* is not.

Sometimes we need to put principles to test theoretically, even if they find little applicability in real-world practice. The disability discrimination problem, thus, does not simply express a misunderstanding of practical economics but involves a realization that the maximization logic underlying the ideal of cost-effectiveness reaches far into the mud of philosophical ethics. In so far as cost-effectiveness refers to the value of maximizing health effect per cost, and health effect is accounted for in ways where disability has negative impact on the measured outcome, a truly cost-effective allocation of healthcare resources *will* discriminate against people with disabilities, even if only indirectly. Certainly, cost-effectiveness analysis is often used in ways that avoid that discrimination, but this does nothing to change the fact that cost-effectiveness by itself *is* discriminatory. Arguably, the fact that cost-effectiveness discriminates against certain groups in part explains why we are often critical of its use as an allocation principle in practice (Harris 2005).

The disability discrimination problem is thus not a critique of cost-effectiveness analysis as part of the health economists’ tool kit but an objection to cost-effectiveness as a suggested distributive principle. We should interpret this objection not as proof that any use of cost-effectiveness analysis ought to be avoided, but as an indication that we should be careful with its use, because cost-effectiveness qua its sensitivity to effectiveness will discriminate against disabled people. If it did not, it would not be adequately sensitive to effectiveness.

The third and final reason for interest in the disability discrimination problem is that in the absence of a careful exploration of the ethics of disability discrimination, there is no way we can know when such discrimination is justified. Often discriminatory policies are morally wrong, but sometimes they are not—sometimes discrimination is justified (Hellman 2008, 4; Lippert-Rasmussen 2013, 103–105; Moreau 2020, 11). Without enquiring into the ethical details of disability discrimination, this distinction is difficult to make.

Despite its susceptibility to being discriminatory, we typically have good reasons to accept cost-effectiveness as a necessary part of a justified rationing scheme. The main reason is that a prioritization that is not cost-effective involves comparatively inefficient allocation of resources which could have been reallocated to benefit people further. Failure to respect the value of cost-effectiveness thus comes with relevant moral opportunity costs (Ubel et al. 1996). This does not mean that a rationing scheme is unjustified if it is not perfectly cost-effective. There are certainly other things to take into consideration as well. But it does imply that we should prefer, other things being equal, a cost-effective scheme to a cost-ineffective one.

Secondly, I define *discrimination* as treating some person(s) comparatively worse than others because of their (actual or perceived) group membership (Alexander 1992; Arneson 2006; Eidelson 2015; Lippert-Rasmussen 2013, 15–26; Moreau 2010; Segall 2012). While standard, this is a generic and non-normative definition—it captures all kinds of differential treatment based on group membership and does not say when and why such differential treatment is wrong. The definition thus needs further normative qualification in terms of what makes discrimination wrong, when it is. Philosophers disagree on this normative part. Some define wrongful discrimination in reference to the disrespect expressed in not treating people as of equal moral worth (Hellman 2008; Eidelson 2015), while others determine wrongfulness in reference to comparative harm (Lippert-Rasmussen 2013); Segall 2012). For the purpose of my argument, it should be adequate to note this distinction and move ahead with the broadest possible moralized account, defining wrongful discrimination as differential treatment that either disrespects or harms the victims of discrimination compared to others.

Paradigm cases of wrongful discrimination are examples of direct discrimination, where the victims are discriminated against by explicit reference to their group membership, but the disability discrimination problem often occurs in reference to discrimination in a more indirect form. How exactly to set the two forms of discrimination apart is a matter of some debate, but it is standard to assume that the distinction is both exhaustive and exclusive such that any instance of discrimination is either direct or indirect, and not both. For the purpose of my argument, I shall adopt the following definition of *indirect discrimination*.

## Indirect Discrimination

An action or policy indirectly discriminates against some individual, if and only if, it does not discriminate in any direct form, but does however impose disadvantages on the group of people to which the relevant individual belongs that are disproportionate relative to the benefits that acting in this way confers on others (adapted from Lippert-Rasmussen 2023, 204).

Finally, I use the term *disability* in a generic sense to refer to activity-limiting disadvantages restricting a person’s performance of important life activities (Hartley 2011; WHO 2017). This definition is ambiguous and a topic for debate (see Barnes 2016). Some theorists define disability as disadvantageous in reference to species normality (Buchanan et al. 2000), others in reference to subjective welfare (Kahane and Savulescu 2009), and recently Jessica Begon has defended a plausible account tying disability to limitations in “abilities that individuals are entitled to be able to perform” (Begon 2021, 947). For my purposes, however, little turns on these particular specifications, and I will proceed with the generic definition. Commonsensical examples of impairments—e.g., paraplegia and blindness—would be considered disabilities on this account. My broad definition also implies that the disability discrimination problem might occur for some conditions that we do not normally count as disabilities (e.g., diabetes). This makes no difference for my argument, however, as the problem still occurs for people with disabilities qua their disabilities.

With these qualifications in place, we can now see how the disability discrimination problem occurs in relation to cost-effectiveness. Imagine that with a fixed bundle of resources, we can provide only one of two possible life prolonging treatments:

- **Scenario A**: Treatment T(a) works effectively against condition C(a) for any patient in population A (ten people). Population A has a *low prevalence* of people with disabilities (one person).
- **Scenario B**: Treatment T(b) works effectively against condition C(b) for any patient in population B (ten people). Population B has a *high prevalence* of people with disabilities (five people).

The disability discrimination problem occurs because cost-effectiveness gives us a pro tanto moral reason to prefer scenario A merely based on the difference in prevalence of disabilities, while the moral obligation of non-discrimination suggests that it is impermissible to take this difference into account.

If disability negatively affects health benefits, cost-effectiveness will take that negative impact into account. Firstly, we can consider the impact of the disability on lifespan. After all, many disabilities shorten people’s life-expectancy, and the duration of time spent in any given health state is important for cost-effectiveness. Second, disabilities also have negative impact on life quality, so cost-effectiveness would consider each life year with a disability as less health-benefit producing than a year without disability. That is, even when disability does not shorten lifespan, standard cost-effectiveness would still give pro tanto reason to favour scenario A over B, if each life year with a disability is viewed as less desirable than each year without (Hadorn 1991; Gold et al. 2002; Whitehead and Ali 2010; Dolan et al. 2005; Harris 2005; Hausman 2015).

A QALY calculation can account for the relevant difference in cost-effectiveness between the two cases. A QALY measures health benefit by multiplying the timespan—duration of time spent in a given health state—by health-related quality of life (HRQoL). HRQoL can be based on simple individual evaluation such as visual analogue scale—judging one’s own health on a scale from zero to a hundred—but is often supplemented by immediate comparative evaluations such as standard gambles or time trade-offs. The HRQoL will provide a zero-one coefficient representing what one year in a particular health state is worth (where 0=death and 1=full health), to be multiplied by the duration of time. In other words, the HRQoL provides the information of the life-quality deficiency from full health of a specific health state or disability.

For illustration, assume thus that we can choose between scenario A or scenario B. To recap, in scenario A we offer life prolonging treatment T(a) for condition C(a) to group A consisting of one patient with a disability and nine patients with no disability. In scenario B, we offer life prolonging treatment T(b) for condition C(b) to group B which has five patients with a disability and five patients without. Assume that the treatments will prolong life with fifteen years for patients with a disability compared to twenty life years for patients without a disability. Moreover, each year with a disability (e.g., being paraplegic or blind) is evaluated to a HRQoL score of 0.8 of full health. With this specification, A QALY calculation for comparing the two scenarios would look like this:

It is clear that a standard cost-effectiveness analysis would recommend scenario A over scenario B. After all, it produces a total of thirty-two additional quality-adjusted life years compared to the alternative in scenario B. Many find this unjustly discriminatory. The central objection is that cost-effectiveness analysis incorporates the unrelated, and undeserved, disadvantage of a disability in the assessment of benefits and thereby makes the priority-setting unfair towards the already disadvantaged—this is sometimes called *the linkage problem*, or *the double-jeopardy problem* (Harris 1987; Kamm 2004; Brock 2009; Ottesen 2013). This is a reasonable objection. It displays the already mentioned concern in relation to fairness that grounds the disability discrimination problem.

Many find intuitive that we have special moral reasons to benefit the already disadvantaged (Nielsen 2022), but cost-effectiveness seems here to do the exact opposite, to somehow punish the disadvantaged for their disadvantage. Recall, however, that by refraining from a cost-effective allocation of resources, even when we have good reasons for doing so, we admit to the moral opportunity costs involved in comparatively inefficient prioritization. This may be, all things considered, justified. Cost-effective allocation is not all that matters. But if it matters at all, we need to enquire into the ethics of the disability discrimination problem. We need, that is, to consider when (if at all) indirect discrimination against people with disabilities is justified by reference to the value of a cost-effective prioritization. Here, a distinction between discrimination based on lifespan (quantitative) and discrimination in reference to life quality (qualitative) makes a significant difference for moral justification, or so I argue.

## Quantitative and Qualitative Disability Discrimination

As I indicated in the introduction, cost-effectiveness discriminates against people with disabilities in two different ways according to the negative impact of disability on the two separate parts of a cost-effectiveness. Firstly, cost-effectiveness will systematically give lower priority to people with disabilities, when the disability results in reduced life expectancy. Call this the *quantitative* part of the discrimination. Second, the discrimination problem also has a *qualitative* part, since cost-effectiveness will count each life year with a disability as of lower quality than each life year without. These are two different sorts of differential treatment—the first concerns lifespan, the second life quality. Both the quantitative and the qualitative part of the discrimination is captured in the example illustrated in table 1.

**Table 1:** Standard QALY calculation for distribution of treatment in two scenarios

	People without disability	People with disability	Total QALYs
**Scenario A**	Life Years 9 x 20 = 180 Quality Adjusted (1) = **180**	Life Years 1 x 15 = 15 Quality Adjusted (0.8) = **12**	**192 QALYs**
**Scenario B**	Life Years 5 x 20 = 100 Quality Adjusted (1) = **100**	Life Years 5 x 15 = 75 Quality Adjusted (0.8) = **60**	**160 QALYs**

However, the two types of discrimination call for different justifications. The quantitative part calls for a justification based on lifespan, whereas the qualitative calls for a justification from life-quality. Critics concerned with the disability discrimination problem are not sufficiently attentive to this distinction, they mostly object to cost-effectiveness on general grounds. However, once these two justifications come apart, it is reasonable to ask whether they strike with equal force. Empirical studies indicate that the public does not think this is the case (Damschroder et al. 2005; Kerstein 2019).

At the heart of this reaction, I conjecture, is the following conjunctive intuition: a justified priority scheme ought not discriminate against people with disabilities based on the reduced quality of life as a result of the disability, but it is on the other hand permitted to take into consideration reductions in lifespan. In my case above, we are justified in letting the fact that T(a) produces more life years than T(b) count (at least pro tanto) in favour of Scenario A, but we should not let the life-quality reduction of the patients’ disability influence our decision. Hence, there seems to be an asymmetry in the justification of the two forms of discrimination. Quantitative discrimination seems more justifiable than qualitative discrimination. Call this *the asymmetry intuition*. As mentioned, the intuition finds some public support but is a rare occurrence in the ethical literature.

How can we explain and give philosophical force to the asymmetry intuition? A first response might be that we cannot, and that we should simply throw it off as irrelevant if not an illusion. In one sense, a sceptic could say that lifespan is just a timespan for having quality of life and that quantity is therefore just the multiplicator by which the substance of quality is multiplied. We can see this in that we would not find reason to save a life with zero quality. Hence, the response goes, it seems that if disability has negative effect on quantity as well as quality of life, both effects should be taken into account, which would render the asymmetry intuition false. Note, however, that this by itself implies nothing about the justification of disability discrimination, only that we should not distinguish clearly between lifespan and life-quality effects.^1^

I find this response inadequate. The asymmetry intuition seems plausible in a way that makes relevant a distinction between the moral justification of qualitative and quantitative discrimination. There are a few noteworthy attempts in the literature to ground the asymmetry intuition. None of them, I think, succeeds but they point sufficiently in the right direction to serve as stepping stones. First, consider Frances Kamm’s *Sufficient Only Option Argument*. The upshot of this argument is that when we take each person’s subjective interest as of equal objective moral importance in our priority setting—Kamm refers to this as *sobjectivism*—it is reasonable to judge disabled and non-disabled lives as equally valuable. Kamm gives the following example:

One may reasonably want 0.5 as much as one would want 1 if one could have it. So, for example, given that 0.5 is all that one can have and 0 is very bad, one might reasonably do as much to achieve 0.5 (e.g. spend as much money, suffer as much) as one would do to achieve 1 if one could have it. This is consistent with the willingness to even risk losing 0.5 and falling to 0 for a chance at 1 (Kamm 2004: 235).

Kamm’s example is meant to give force to the intuition that the value of saving a person’s life depends on the option of life available from a subjective point of view. This implies that prolonging the lives of disabled people can reasonably be judged to hold as much moral value as prolonging the lives of the non-disabled, despite that we from an objective point of view consider the latter type of lives preferable for all. Indeed, Kamm notes, the Sufficient Only Option Argument is compatible with disabled people preferring not to have a disability if given the opportunity.

The problem with the Sufficient Only Option Argument is that it merely explains the first half of the conjunction in the asymmetry intuition. While it captures a relevant reason why we should not discount the value of disabled lives, it fails to explain why we should on the other hand allow lifespan considerations. The problem is that just like a disabled person can want ten years of 0.5 life-quality just as strongly as she would have wanted ten years of 1 life-quality had it been an option, a disabled person can similarly want five years of life as strongly as she would have wanted ten years of life had *that* been an option (Harris 2005). Consequently, the Sufficient Only Option Argument only takes us halfway to our destination.

Kamm anticipates this and for that reason turns to the *Equal Respect Argument*. According to this argument, we owe everyone equal respect and concern, which implies not discriminating based on (at least certain) synchronic properties, constitutive of a person’s identity or character of life. The Equal Respect Argument captures the asymmetry intuition because the qualitative aspect of a disability is (or at least can be) a synchronic property, whereas lifespan is not. Kamm explains:

However, taking into account, for example, how long a person can live if he gets a scarce resource is not treating someone differently because of the type of person he is or will be qualitatively; the latter (it is being suggested) is done only if we consider someone’s synchronic properties (properties that determine the character of his time alive). It is, theoretically, compatible with each synchronic type that a person could be, that he could be that type for longer or shorter amounts of time (Kamm 2009: 168).

The Equal Respect Argument captures both sides of the conjunction in the asymmetry intuition. But it faces several problems. One problem is that it is unclear about, firstly, what counts as synchronic properties and what doesn’t and, secondly, which synchronic properties we are not allowed to consider. We understand that disability is one category of synchronic properties and that differential treatment based on this category would be disrespectful, but without further justification, this seems ad hoc at best, circular at worst. It is ad hoc if we are not offered an account of why disability, but not other synchronic properties, is relevant. It is circular if the explanation for why we take disability into account already presupposes that differential treatment based on disability is disrespectful.

A second problem concerns the assumed connection between respect and the demand to not treat people unequally based on synchronic properties. Since we can imagine cases where differential treatment based on (what seems to be) non-synchronic properties is also intuitively disrespectful, the connection is drawn into question—Samuel Kerstein discusses the case of differential treatment based on blood types as an example (Kerstein 2017).

As an alternative to Kamm’s Equal Respect Argument, Samuel Kerstein provides a Kant-inspired account to explain the asymmetry intuition not by reference to the value *for* persons but to the value *in* them. Kerstein’s account invokes a Kantian view on human dignity based on the special status of personhood and therefrom states that in the spirit of respect for human dignity, we ought to treat any person as having “unconditional, preeminent worth” (Kerstein 2017). On Kerstein’s view, offering treatment to A but not B, on the expectation that B will have lower health-related quality of life than A, fails to respect that both have unconditional, preeminent worth. But, secondly, Kerstein’s account can incorporate lifespan in the assessment because of his *Preservation Argument* (my label). The Preservation Argument says that (i) personhood has special (unconditional, preeminent) value and (ii) acting with respect for the special value of things often involves trying to preserve them (Kerstein 2017, 646–647). The upshot of the argument is that, since we have reasons to maximize preservation of personhood, and thereby personhood years, we should make healthcare priorities sensitive to lifespan while insensitive to differences in health-related quality of life. This, Kerstein would say, is a more promising route to the justification of the asymmetry intuition.

While I am sympathetic to Kerstein’s view, I think we have reasons to reject the Preservation Argument. My central reason for rejecting it is that I don’t think switching from the *value for* to the *value in* people well explains our life-prolonging duties. What matters for Kerstein is that respect for personhood is the appropriate way to acknowledge the intrinsic value of human worth, but the link from here to a duty to preserve “personhood years” is underdeveloped and theoretically fragile. Kerstein has not shown that prolonging lifespan follows from respecting personhood. If Kerstein is right, we would have no problem accepting highly paternalistic restrictions on people’s imprudent life choices (e.g., smoking or engaging in extreme sports) because these are effective ways to preserve personhood years. However, most find that the problem with paternalistic policies, even when all-other-things-considered justified, is exactly that they disrespect personhood. Moreover, in some instances, we even think that the best we can do to respect personhood is to not preserve it. For these reasons, I think we should look for an alternative justification for caring about lifespan. In the remainder of this paper, I provide an alternative argument which I think better accounts for the asymmetry intuition.

## The Reasonable Impartial Interest Argument

My argument takes inspiration from Kamm and Kerstein’s general views, yet at the heart of my argument is the moral salience of *reasonable impartial interest*. The argument has three premises, and I will elaborate or defend each of them in turn. From these three premises, it follows that quantitative is more justifiable than qualitative disability discrimination when considering cost-effectiveness as part of a justified scheme for healthcare priority setting.

(P1) In setting healthcare priorities, it is, other things equal, important that resources are used effectively to produce as much benefit as possible (*The Beneficence Claim*).

As is clear by now, the beneficences claim implies cost-effectiveness, when there are budget constraints, and I think this is controversial. As mentioned, some critics might object that cost-effectiveness is often morally suspicious—i.e., leads to unfair outcomes—but few would deny that a justifiable scheme for healthcare priority setting should pro tanto take cost-effectiveness into account. Moreover, the “other things equal” clause makes the beneficence claim appropriately weak to imply that other relevant factors may, under given circumstances, outweigh our reasons for a cost-effective allocation. Note also that if we deny the beneficence claim, the disability discrimination problem never occurs. Thus, the central point at this stage is merely that if you agree with the first premise as stated, we have reasons to proceed with how to make assessments of cost-effectiveness.

The next premise says something about how health benefits should be counted.

(P2) In the assessment of cost-effectiveness, health benefits should be included in accordance with a criterion of reasonable impartial interest (*the Reasonable Impartial Interest Claim*).

This premise needs to be defended. A cost-effectiveness analysis accounts for health-related benefit per cost, and so an account of relevant health benefits is needed. The first thing to note is that the individual is the proper entity for counting relevant interests in this context. It would seem misplaced, as a way of measuring the relevant value of health, to take a collective perspective—e.g., the overall economic or social interest of a society. Health benefits are valuable because of the way they benefit the individual.^2^

Not all individual interests should count, however. In assessing cost-effectiveness of some suggested healthcare priority setting, it would be unjustified to take into consideration the effect of contingent health-unrelated factors on a person’s life. It would be unjustified, for example, if a prioritization scheme deciding whether or not to save my life considered irrelevant factors—like the fact that I have fewer siblings than others, or that my personal passions, such as swimming with whales, are less likely to be fulfilled compared to other patients’ interests. These are factors of central importance for individual persons’ interests in life, but which should be filtered out of the assessment of relevant health benefits. The most important reason for that is the objective moral importance of health needs. A central moral pillar in a healthcare system is the assumption that we are all, to some extent, interested in being protected against having unmet health needs. While health needs are indeed very individual—and personalized medicine has helped us understand this even more—the life-course need for basic healthcare is an objective interest (Daniels 2008; Schramme 2018). It is, like Rawls’ social primary goods (Rawls 1999), something we all have reason to value despite our otherwise subjective preferences.

Moreover, while personal reasons to value health benefits are partial—your reasons to want a hip replacement are indeed exclusively yours—the reasons involved in the comparative assessment of different people’s competing interest in the same health benefit should be justifiable across individual partiality. If reasons to discriminate against patients in population B in comparison to A should be factored into our cost-effectiveness analysis, these reasons should be justifiable from the perspective of both A and B patients. Importantly, this does not imply that only a completely consensus-based prioritization is acceptable. Rather, and more modestly, it implies that the factors included in the assessment of health benefits should be reasonably acceptable regardless of any individual’s particular perspective. This claim to impartiality is a key feature in contemporary contractualist accounts (Scanlon 1998; Dworkin 2000; Daniels 2008) but can also be justified without contractualist foundations (Raz 2003). It becomes even more prominent in publicly funded healthcare systems, because political priorities should be reasonably justifiable to all contributors, regardless of their partial interest in the outcome.

At this point, it should be clear that we are justified in our use of cost-effectiveness analysis, and that relevant health benefits should be assessed using a criterion of reasonable impartial interest. The last premise of the argument basically reiterates the asymmetry intuition and links that to the premise of reasonable impartial interest.

(P3) On a criterion of reasonable impartial interest, it would be more justifiable to discriminate based on differences in lifespan than to discriminate based on judgements regarding life quality. (*The Asymmetry Claim*)

The key argument for this final premise is that we have less (if any) adequate basis for making judgements on life quality—and hence neither on the potential negative impact of disability on life quality—from an impartial perspective, but that we can, on the contrary, meaningfully make impartial judgements on the importance of lifespan. Let me unfold this defence in some detail.

Imagine a rescue case, in which we can rescue only one of two patients, P or Q. In such a rescue case, it does not matter from an impartial perspective who we rescue. If we can choose between rescuing P or Q, we find ourselves, pro tanto, in a tie with equally strong reasons to rescue both. This does not imply that P and Q necessarily have the same partial interests in their life. Plausibly, P and Q each have their individual dispositions, life plans, and preferences, and it would be unreasonable to expect these to have the exact same impact on P and Q’s quality of life. But the central point is that we have no authority to make judgements on the importance of these interests. Even if we had full knowledge of P and Q’s complete set of personal preferences, we are in no position to judge how they affect their interest in life. Indeed, even if we assume that P and Q have the exact same life-plans and prospects—say, to combine a career as a primary schoolteacher with taking care of a family of four—it is beyond our authority to judge how that matters to their general interest in life. What is implied, thus, is that from an impartial perspective we have no adequate basis for weighing these partial interests against each other. All we can do is to acknowledge that, in part because P and Q each have their own partial reasons to value life, we have equally strong *impartial* reasons to value life *for them*.

This part of the argument reinforces Kamm’s Sufficient Only Option Argument. To reiterate, Kamm’s argument says that just like we have no authoritative reason to discount the interest in life of either P or Q, so we have no authority to discount the interest of disabled people in a life with disability. My Reasonable Impartial Interest Argument complies but adds that because individuals are the proper ethical entity for our assessment, and because individuals’ interest in the lifetime they have is always determined from a partial perspective, we have no authority to discount anyone’s general interest in life from an impartial perspective. Where Kamm suggests that a one person’s interest in a life of 0.5 health-related quality is just as strong as a another person’s interest in a life in full health, my arguments adds that once we acknowledge that our impartial assessment concerns the interest of individuals in life *for them*, there is in fact no reasons to trust that a life with disability is in any meaningful sense of lower value than any other life.

Critics might object that many disabilities do make life worse in ways that can be acknowledged from an impartial perspective. Being unable to move one’s legs is worse for anyone in that position and is thus different from contingent subjective interests. This objection misses the point. The reasonable impartial interest claim only implies that people’s interest in the life that they are given should be counted equally from an impartial perspective. This is compatible with recognizing that disabilities are unwanted disadvantages. The claim is not the disabilities are not bad for people, or that people are wrong in wanting to recover from disabilities, but that we have no authoritative way of impartially judging how, or even if, that disadvantage affect their interest in life.

Now, the final small step in the defence of the asymmetry claim is to accept that there is no morally relevant difference between a rescue case and a case of allocating life-prolonging treatment. This is uncontroversial. There seems to be no meaningful sense in which rescuing someone’s life is not the same as prolonging their life. If I can rescue only P or Q from death, it follows that I can also only prolong either P or Q’s life (and vice versa). But if this is true, it seems that prolonging life serves a parallel purpose in our moral deliberation as numbers count in rescue cases, at least in the following way. To adopt a framework from Raz (2003), we have impartial moral reasons to rescue anyone who can be rescued, and full compliance with these reasons requires rescuing everyone. When it is impossible to rescue everyone (who can be rescued), we are complying to a larger degree with our moral reasons, the more we rescue of those who could be rescued. This is, I take it, a plausible framework of how numbers count from an impartial perspective.^3^ If numbers count in this way, and rescuing is not relevantly different from prolonging life, we should consider timespan differences relevant. If I can prolong P’s life with N or Q’s life with N-10, it follows that my situation involves a tragic choice in the sense that I cannot possibly comply fully with my impartial moral reasons—i.e., my reasons to prolong both P and Q’s life. But it also follows that I comply to a larger extent with these impartial reasons if I choose to prolong P’s life with N rather than Q’s life with N-10. This explains why timespan should matter as an impartial reason. Coming back to my original case, the Reasonable Impartial Interest Argument implies that differential treatment of patient population A and B is more justifiable if based on the reason that Scenario A will produce *more* life years than Scenario B, than if based on the questionable judgement that it will produce *better* life years. One implication of this could be to turn to health value comparisons that are insensitive to variations in life-quality.^4^

An analogy might help illustrate the general line of thought in the asymmetry claim. Imagine two children highly and equally interested in playing the piano, and say that we can give a piano to only one of them. If given the piano, the first child can play for ten additional years, whereas the second child can play for only one year. I have the intuition that we are justified in giving the piano to the first rather than the second child, and I think a justification for this prioritization can be given without unreasonably discounting the interest of the second child. The justification is that since timespan playing piano is equally valuable for each of them, ten years of piano playing is much more—sobjectively, we might add—valuable than one year playing piano, even when we count their interest in piano time as of equal moral importance. In making this judgement, I am not asserting anything about their piano skills. Adding that one of them is better capable of playing the piano makes little difference, if their interest in piano time is equally strong. In a parallel way, I would hold, since timespan living is of equal, impartial interest to persons with and without disabilities, variations in lifespan is a salient, relevant moral factor even when we count equally their individual interest in living the life they have available.

At this point, critics might object that my argument seems to imply that we should always maximize life-prolonging effects. But this does not follow necessarily. My argument only implies that timespan matters when considering justifiable cost-effectiveness assessment. It does not imply that it is all that matters or that it should count with any specific weight. Age-sensitive accounts (Daniels 1993; Bognar 2015; Nielsen 2021) suggest that we have stronger, impartial, reasons to value treatment for the young than for the elderly—e.g., related to lifespan fairness. My argument is fully compatible with this. It only implies that it would be more justified to include reasons related to difference in timespan than assessments of life quality.

As a final qualification, note that my third premise is formulated rather weakly. The asymmetry claim only says that it is *more* justified to discriminate based on timespan than on judgements regarding life quality. Hence, the premise can be true even if it turns out that neither qualitative nor quantitative discrimination is justified. It follows that the Reasonable Impartial Interest Argument is, at least in principle, open to both advocates for and critics of cost-effectiveness. Even if, for example, the critics are right and it turns out that we have strong reasons to never use cost-effectiveness analysis in a way that discriminates against disabled people, my argument implies that qualitative discrimination is still more morally problematic than quantitative discrimination.

To conclude, the Reasonable Impartial Interest Argument seems better equipped to account for the asymmetry intuition than existing views. First, it elaborates upon and adds further to the key contribution of Kamm’s Sufficient Only Option Argument, and second, it explains why timespan considerations are morally relevant from an impartial perspective, despite that judgements on life quality are not. Much more need to be uncovered. My central aim was to break open the overlooked issue of the asymmetry between lifespan and life-quality. If my argument does not take us all the way, we need further philosophical inquiry, and it might turn out that there is no sound way to defend the intuition. But the Reasonable Impartial Interest Argument is, in my view, the strongest contender for providing the needed foundation, and I have explained why I think it does a better job than Kamm and Kerstein’s accounts. This, at least, takes us a further step towards uncovering the ethics of disability discrimination.

## Conclusion

This paper introduced and defended the asymmetry intuition, that it is more justifiable to discriminate against people with disabilities based on lifespan considerations than on their lower health-related quality of life. As a philosophical exploration of the ethical foundation for this intuition, the paper discussed Frances Kamm’s Sufficient Only Option Argument and Samuel Kerstein’s Kant-inspired dignity account. I argued that while both serves to guide us in the right direction, none of them succeeds in adequately accounting for the intuition. As an alternative, I offered the Reasonable Impartial Interest Argument, that because cost-effectiveness assessments should be made from an impartial perspective, and because timespan differences matter for our impartial moral reasons in a way that life-quality judgements do not, quantitative disability discrimination is more justifiable than qualitative disability discrimination. For practical purposes, this implies that cost-effectiveness analysis should be careful with quality-adjusted assessments based on disabilities but is more justified in taking expected life year benefits into consideration. This conclusion contributes to the continuing ethical discussion of the disability discrimination problem—in particular, by pointing to a way of embracing cost-effectiveness without unjustly discriminating against people with disabilities based on allegedly life-quality reducing effects of their disability.

## Data Availability

Not applicable (no original data)

## Footnotes

1 Singer et al. (1995) reject the distinction and argue that disability discrimination is not unjustified, whereas Harris (2005) similarly rejects the distinction but argues against the justification of disability discrimination.

2 This is compatible with different accounts of the value of health (see Hausman 2015).

3 A similar argument could be derived from Scanlon (1998) or Kumar (2001).

4 For such accounts, see Nord et al. (1999); Aas and Wasserman (2017: 178); Nielsen (2023).
